# The Late Baron Berzelius

**Published:** 1848

**Authors:** 


### ARTICLE IX.

### *The late Baron Berzelius.*

This distinguished chemist, the father of analytical chemistry, expired on the 7th of August, at Stockholm. Baron Berzelius was born on the 20th of August, 1779, Ostergothland, in Sweden, of a respectable family. At the age of 17 he entered the University of Upsala, where he made rapid progress in his studies, particularly his favorite science—chemistry; after passing the necessary examinations, he received his diploma of Doctor in Medicine in 1804, and was appointed Medicinæ et Pharmaciæ Adjunctus at the Collegium Medicum at Stockholm, and gave instructions in chemistry to young students and, on account of his small income, was obliged to practise occasionally as a physician. In the year 1807 he was appointed Medicinæ et Pharmaciæ Professor, and in the same year he instituted, in conjunction with seven other eminent men, the Swedish Medical Society at Stockholm, now a most flourishing institution, and constituting the very heart of the medical profession in Sweden.

In 1808 he was made a member of the Royal Academy of Science, in 1810 officiated as president, and in 1818 as perpetual secretary. On the occasion of holding this appointment for a quarter of a century, a dinner was given in the Academy by the members to this distinguished *savant*, which was presided over by his present majesty, then the crown prince, who, on proposing the health of Berzelius, expressed his grateful acknowledgment of his own obligations to Berzelius for the valuable private instruction he had received from him in his younger days. In the same year he was appointed a member of the Royal Sanitary Board, of which, at the time of his death, he was the senior member. As a proof of the magnitude of his laborious pursuits, it may be sufficient to mention that he first developed the electro-chemical system, and that he has also examined and minutely described the atomic theory of the elementary bodies. He discovered and examined several great classes of chemical combinations, as, for instance, the different degrees in which sulphur combines with fluoric acid, with platinum, columbium, vanadium, tellurium and phosphorus, the sulphates, &c. In organic chemistry he has no less distinguished himself by his experiments; and, properly speaking, he has laid the foundation of vegetable and animal chemistry; more particularly the latter. As regards chemical analysis, the highest merits are due to him, for having arranged a new and generally-adopted chemical nomenclature. His works, which have been for the most part translated into the English, French, German, Italian, Spanish and Polish languages, are so numerous and voluminous that, considering the accuracy with which everything is described, it appears to be almost a wonder how one man, whose time, besides, is occupied by a great amount of official duties, has been able to accomplish such a mass of scientific publications.

Berzelius had received from his Majesty King Charles John many marks of high distinction: he was created a nobleman in 1818, a Baron in 1835, Knight Commander of the Royal Order of Wasa in 1821, and Grand Cross of the same order in 1829; he was a Knight of the Royal Swedish Order of the Polar Star, and of several foreign orders received from the Emperor of Russia and the Kings of Prussia, Denmark, Belgium, France and Sardinia; an honorary member of upwards of one hundred literary and scientific societies. In consideration of the great services which Berzilius had bestowed on his native country, the members of the Diet at Stockholm, in 1840, voted to him the annual sum of 2000 dollars banco as a pension for his lifetime, independent of his former emoluments.

The Edinburgh Journal of Science and the Philosophical Magazine, to which we are indebted for these particulars, state, as showing how high the illustrious deceased ranked in

Germany, that in a late history of the Devil, of which so many are published in that country, one of the main inducements his Satanic majesty is represented as holding out to a convert, still half doubtful of selling himself, is, that he will make him *a Berzelius.—London Lancet.*

